# A Second Trimester Caesarean Scar Pregnancy

**DOI:** 10.1155/2014/828635

**Published:** 2014-03-23

**Authors:** Pooja Sikka, Vanita Suri, Seema Chopra, Neelam Aggarwal

**Affiliations:** ^1^Post Graduate Institute of Medical Education and Research, Chandigarh, India; ^2^Department of Obstetrics and Gynaecology, PGIMER, Chandigarh 160012, India

## Abstract

Caesarean scar pregnancy, where conceptus is implanted on previous scar, is a rare entity. We present one such case of scar pregnancy presenting to us in the second trimester and was managed with methotrexate and uterine artery embolization, followed by hysterotomy. Uterus could be conserved and hysterectomy could be avoided.

## 1. Introduction

Caesarean scar pregnancy, where conceptus is implanted on previous scar, is a rare entity. However, in recent years, there have been several reports on first trimester diagnosis of such pregnancies. Availability of high resolution transvaginal sonography and its increasing use in early gestation has resulted in early and more frequent diagnosis of this condition. Commonly scar pregnancy presents as threatened, incomplete, or complete abortion in the first trimester. Occasional pregnancies may progress to second and third trimester and develop into placenta previa/accreta. Currently there are no guidelines for the management of such pregnancies. We present one such case of scar pregnancy presenting to us in the second trimester and the difficulties in her management.

## 2. Case Report

A 25-year-old gravida 3, para1 was admitted at 19 weeks of pregnancy with a history of vaginal bleeding for 1 week and pain in abdomen. She was told that she had a low lying placenta during her second trimester scan. A lower segment caesarean section for cephalopelvic disproportion was done 2 years back and the baby was alive and well. This was followed by a missed abortion at 8 weeks for which a curettage was done. At admission, her vitals were stable. Uterus was irritable. Hb was 7 gm/dL. Ultrasound done after admission showed a live fetus of 20 weeks and anterior placenta with a thin, bulging, and deficient lower uterine segment. The decidual interface between the placenta and myometrium was partially absent and there were large dilated vessels in the same area. These sonographic features were suggestive of a placenta accreta. Patient continued to bleed; 4 units of blood were crossmatched and injection of methotrexate 50 mg was given intramuscularly on the day of admission. Prophylactic uterine artery embolisation was done on day 2 but the bleeding continued. The next day she was taken up for hysterotomy under general anaesthesia. Entry into peritoneal cavity was difficult because of dense adhesions. There was no hemoperitoneum. Bladder was adherent to the lower uterine segment which was severely deficient. Placenta was encroaching on the left broad ligament and was covered by a thin layer of peritoneum. Bladder was dissected from the lower uterine segment and incision was given at the previous scar. Fetus was extracted out first and placenta was then clearly seen to be firmly adherent to myometrium at several sites. Most of it could be removed piecemeal. Hemorrhage was controlled by uterotonics and hemostatic sutures at the placental bed. The estimated blood loss was approximately 1 litre. Two units of blood were transfused intraoperatively. Her postoperative Hb was 8 gm/dL. Catheter was removed on day 5. Recovery was uneventful. She was discharged 1 week later on iron tablets and contraceptive advice.

## 3. Discussion

Caesarean scar pregnancy is the rarest form of ectopic pregnancy. However, with rising rate of caesarean deliveries over the world, probably its incidence would increase. Thus, it is important to have a high index of suspicion in patients with risk factors. Scar pregnancy is difficult to differentiate from a cervical pregnancy. Cervical pregnancies rarely progress to term, whereas scar pregnancies may do so because of their position at the level of internal os. Also, several diagnostic criteria of caesarean scar pregnancy have been described in literature. The diagnosis is usually made on ultrasonography which shows empty uterine cavity and an empty cervical canal, gestational sac in the anterior part of the uterus, and absence of healthy myometrium between bladder and sac [1]. There are no universal treatment guidelines for caesarean scar pregnancy. Due to relative rarity of scar pregnancy, it is still unclear which treatment is most optimal. The diagnosis and management of any pregnancy implanted over a scar is not difficult in the 1st trimester, because the gestational sac is very small and the lower segment is thick enough that a defect in the scar can be seen. A 1st trimester diagnosis of abnormal placentation can give women the option to choose between expectant management and termination of pregnancy. Many authors advocate that all scar pregnancies should be terminated once their diagnosis has been made. The main management options are expectant, surgical, and medical. The reported results of expectant management are variable with only a few successful cases [2, 3]. Medical management mainly consists of methotrexate, given either intramuscularly or locally. Though an efficacy of 80% has been reported, the safety of medical management is still unknown [4]. Despite a falling *β*-hCG level, bleeding and rupture may still occur in a scar pregnancy that is managed medically [4]. Dehiscence and repeat scar pregnancy has been reported after local methotrexate treatment [5]. Prophylactic bilateral uterine artery embolization has also been employed by some to minimize heavy bleeding [4]. In 7 women who were managed expectantly either by patient choice or because of wrong diagnosis, the hysterectomy rate was 70% [1, 2, 5–7]. In our case, the patient initially had nonsurgical management with a combination of intramuscular methotrexate and uterine artery embolization. Fetus was still viable and vaginal bleeding continued. Hysterotomy was resorted on day 3 of admission in fear of scar rupture/uncontrollable haemorrhage. We assume that combined modalities (both nonsurgical and surgical) employed in this patient may have minimized the complications of heavy bleeding and thus the need for hysterectomy. The possibility of conserving the uterus is important to women who have not completed their families as was our patient. These patients should be offered surgical repair of the scar either as primary treatment or as a secondary operation after initial treatment [8]. Even in the 1st trimester, risk of haemorrhage is 20%–40%. The risk of resorting to hysterectomy is low if internal iliac ligation is performed first.

Our case shows that a successful diagnosis of placenta previa/accreta developing within a deficient scar can be made in the 2nd trimester also. Administration of methotrexate and uterine artery embolization to reduce the placental circulation can cut down the blood loss during surgery. Accurate diagnosis helps to ensure that senior obstetricians are present at the time of intervention so that chances of surgical complications, need for hysterectomy, and death may decrease. We advocate that all scar pregnancies should be reported so that there is more information on diagnosis, safety, and efficacy of various treatment modalities.
